# The prevalence of anxiety and depression in transgender people living in russia

**DOI:** 10.1192/j.eurpsy.2021.449

**Published:** 2021-08-13

**Authors:** E. Chumakov, Y. Ashenbrenner, N. Petrova, M. Zastrozhin, L. Azarova, O. Limankin

**Affiliations:** 1 Day Inpatient Department, St-Petersburg Psychiatric Hospital No 1 named after P.P. Kaschenko, Saint-Petersburg, Russian Federation; 2 Department Of Psychiatry And Addiction, Saint-Petersburg University, Saint-Petersburg, Russian Federation; 3 Department Of Addictology, Russian Medical Academy Of Continuous Professional Education, Moscow, Russian Federation; 4 Laboratory Of Genetics And Basic Research, Moscow Research & Practical Centre on Addictions of the Moscow Department of Healthcare, Moscow, Russian Federation; 5 Department Of Psychotherapy, Medical Psychology And Sexology, North-Western State Medical University named after I.I. Mechnikov, Saint-Petersburg, Russian Federation; 6 Department Of Social Psychiatry And Psychology, St-Petersburg Institute of Postgraduate Improvement of Physicians-experts of the Ministry of Labour and Social Protection, Saint-Petersburg, Russian Federation

**Keywords:** Depression, Transgender, Anxiety

## Abstract

**Introduction:**

The prevalence rates of mental health issues, particularly anxiety and depression, is high among transgender people. However, the incidence of anxiety and depression in transgender people living in Russia is unclear until now.

**Objectives:**

To examine the frequency of anxiety and depression in transgender people living in Russia.

**Methods:**

The Hospital Anxiety and Depression Scale (HADS) was used for online screening for symptoms of anxiety and depression in transgender people living in Russia throughout November 2019. 588 transgender adults living in all Federal Districts of Russia (mean age 24.0±6.7) were included in the final analysis. 69.6% (n=409) of the survey participants indicated the direction of transition as transmasculine (TM), 23.1% (n=136) – as transfeminine (TW), and 7.3% (n=43) – as other (TO).

**Results:**

It was found that 45.1% (n=265) and 24.0% (n=141) of transgender people had clinically significant levels of anxiety and depression, respectively (HADS score of 11 or higher). The rates of anxiety (TM=10.21±4.68; TW=8.72±3.91; TO=10.72±4.43) and depression (TM=7.53±4.09; TW=7.40±4.19; TO=7.74±4.33) did not have statistically significant differences within the direction of transition. The anxiety and depression mean scores in all subgroups were statistically significantly higher than in the general Russian population (p<0.001; one sample t-test).

**Conclusions:**

Our findings suggest a high prevalence of depression and anxiety disorders in the transgender population as compared to the cisgender population in Russia. The identified frequency of anxiety and depression in transgender people in Russia is worrying and requires immediate action to improve the availability and quality of medical and psychological care for this group of people.

**Disclosure:**

No significant relationships.

